# Fluoroscopically-guided interventions with radiation doses exceeding 5000 mGy reference point air kerma: a dosimetric analysis of 89,549 interventional radiology, neurointerventional radiology, vascular surgery, and neurosurgery encounters

**DOI:** 10.1186/s42155-020-00159-6

**Published:** 2020-09-22

**Authors:** Jacob J. Bundy, Ian W. McCracken, David S. Shin, Eric J. Monroe, Guy E. Johnson, Christopher R. Ingraham, Kalpana M. Kanal, Richa A. Bundy, Sean T. Jones, Karim Valji, Jeffrey Forris Beecham Chick

**Affiliations:** 1 Wake Forest Baptist Health, One Medical Center Boulevard, Winston-Salem, NC 27157 USA; 2 University of Washington, 1959 Northeast Pacific Street, Seattle, WA 98195 USA

## Abstract

**Purpose:**

To quantify and categorize fluoroscopically-guided procedures with radiation doses exceeding 5000 mGy reference point air kerma (K_a,r_). K_a,r_ > 5000 mGy has been defined as a “significant radiation dose” by the *Society of Interventional Radiology*. Identification and analysis of interventions with high radiation doses has the potential to reduce radiation-induced injuries.

**Materials and methods:**

Radiation dose data from a dose monitoring system for 19 interventional suites and 89,549 consecutive patient encounters from January 1, 2013 to August 1, 2019 at a single academic institution were reviewed. All patient encounters with K_a,r_ > 5000 mGy were included. All other encounters were excluded (*n* = 89,289). Patient demographics, medical specialty, intervention type, fluoroscopy time (minutes), dose area product (mGy·cm^2^), and K_a,r_ (mGy) were evaluated.

**Results:**

There were 260 (0.3%) fluoroscopically-guided procedures with K_a,r_ > 5000 mGy. Of the 260 procedures which exceeded 5000 mGy, neurosurgery performed 81 (30.5%) procedures, followed by interventional radiology (*n* = 75; 28.2%), neurointerventional radiology (*n* = 55; 20.7%), and vascular surgery (*n* = 49; 18.4%). The procedures associated with the highest K_a,r_ were venous stent reconstruction performed by interventional radiology, arteriovenous malformation embolization performed by neurointerventional radiology, spinal hardware fixation by neurosurgery, and arterial interventions performed by vascular surgery. Neurointerventional radiology had the highest mean K_a,r_ (7,799 mGy), followed by neurosurgery (7452 mGy), vascular surgery (6849 mGy), and interventional radiology (6109 mGy). The mean K_a,r_ for interventional radiology performed procedures exceeding 5000 mGy was significantly lower than that for neurointerventional radiology, neurosurgery, and vascular surgery.

**Conclusions:**

Fluoroscopically-guided procedures with radiation dose exceeding 5000 mGy reference point air kerma are uncommon. The results of this study demonstrate that a large proportion of cases exceeding 5000 mGy were performed by non-radiologists, who likely do not receive the same training in radiation physics, radiation biology, and dose reduction techniques as radiologists.

## Introduction

Minimally invasive, image-guided interventional procedures have expanded the scope of medical practice across numerous domains of medicine, owing to the demonstrated benefit to patients (Heilmaier et al. 2015). As the utilization of these procedures increases, the health risks associated with ionizing radiation to the patients and physicians must be given further consideration (Koenig et al. 2001; Jaschke et al. 2017). The radiation dose between fluoroscopically-guided procedures may vary significantly, and in an effort to reduce patient radiation exposure, radiation dose displays have been required on fluoroscopy equipment (International Electrotechnical Commission 2010; Food and Drug Administration, HHS 2005).

Radiation dose is traditionally measured using fluoroscopy time (FT), dose-area-product (DAP), also known as kerma-area-product (P_KA_), and reference point air kerma (K_a,r_). Stochastic effects of radiation, including carcinogenesis and hereditary effects, are estimated using DAP. The deterministic health effects, which vary with the dose of radiation and include skin injury, hair loss, and cataracts, are assessed using peak skin dose (PSD) or K_a,r (__Bundy et al._ 2018_;__Duncan et al._ 2013_)_. In 2009, the *Society of Interventional Radiology* (SIR) set forth recommendations regarding radiation dose management related to interventional radiologic procedures (Stecker et al. 2009). These guidelines defined a significant radiation dose threshold as any of the following: PSD > 3000 mGy, K_a,r_ > 5000 mGy, DAP > 500 Gy·cm^2^, or FT > 60 min (Stecker et al. 2009). These thresholds were established to identify patients who require clinical follow-up for potential deterministic radiation-induced injury.

The varying complexity of pathologies treated using fluoroscopically-guided endovascular procedures leads to a significant number of prolonged procedures, which results in increased radiation exposure to patients and operators (Hassan and Amelot 2017; Kirkwood et al. 2015; Miller et al. 2003). Identifying those procedures associated with significantly high radiation doses will allow for more tactical dose management strategies that may reduce the likelihood of radiation injury to patients and limit the cumulative radiation exposure to physicians.

The purpose of this study was to quantify and categorize fluoroscopically-guided interventions with K_a,r_ exceeding 5000 mGy.

## Materials and methods

### Study design

This study was conducted with *Institutional Review Board* approval and complied with the *Health Insurance Portability and Accountability Act of 1996*. Radiation dose data were recorded and extracted using dose management software (DoseWatch; GE Healthcare, Buc, France) which was installed in all angiography suites. The DoseWatch software captured dosimetric data from 19 interventional suites including 4 hybrid operating rooms. Radiation dose data for 89,549 consecutive patient encounters from January 1, 2013 to August 1, 2019 at a single academic institution were reviewed.

### Inclusion and exclusion criteria

All patient encounters with K_a,r_ > 5000 mGy were included (*n* = 260). All other encounters were excluded (*n* = 89,289). Each intervention was treated as a single patient encounter. Patients who underwent more than one intervention in separate sessions were included as discrete encounters.

### Collected and defined parameters

Patient demographics, medical specialty, intervention type, FT, DAP, and K_a,r_ were evaluated. Medical specialties included interventional radiology, neurointerventional radiology, neurosurgery, or vascular surgery. FT was defined as the total time that fluoroscopy was used during the intervention and was recorded in 0.1-min increments. DAP was defined as the product of the dose in air in a given plane by the area of the entire x-ray beam emitted from the x-ray tube. K_a,r_ was defined as the air kerma accumulated in space relative to the fluoroscopic gantry. All dosimetry parameters were automatically recorded by each fluoroscopic unit per industry standards (International Electrotechnical Commission 2010; Jones and Pasciak 2011; NCRP Report 168 | NCRP | Bethesda, MD n.d.).

### Patient demographics

Patient demographics are shown in Table 1. Demographic and dosimetric data were collected for 260 discrete encounters. The study included 159 (61.2%) male subjects, 80 (30.8%) female subjects, and 21 (8.1%) subjects of unknown sex. Mean age was 61 ± 17 years (range: 18–94 years). There was no significant difference in demographics between medical disciplines.

**Table 1:**
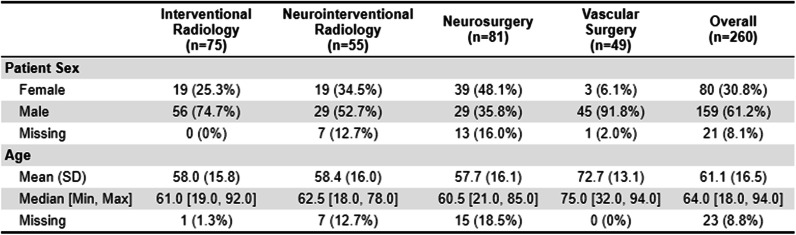
Patient demographics. Statistical significance level is *P* < 0.05. (SD = standard deviation)

### Statistical analyses

Statistical analysis and graphic representations were derived using R software version 3.2.2 (R Core Team; Vienna, Austria). The mean FT, DAP, and K_a,r_ were obtained by summing the values for each individual procedure within each medical discipline and dividing by the total value from all procedures within the discipline. DAP and K_a,r_ were normalized prior to parametric testing. Independence between two categorical variables was assessed by Fischer’s exact test or one-way ANOVA. Two-sample t-tests were conducted to compare population means of two independent groups. During pairwise testing, *p* values were adjusted using the Benjamin and Hochberg correction. A *p*-value < 0.05 was considered statistically significant for all tests.

## Results

During the study period, 260 (0.3%) discrete fluoroscopically-guided procedures had a K_a,r_ which exceeded 5000 mGy. Table 2 displays the distribution of procedures by medical discipline and the mean FT, DAP, and K_a,r_. Of the 260 procedures which exceeded 5000 mGy, neurosurgery performed 81 (30.5%), followed by interventional radiology (*n* = 75; 28.2%), neurointerventional radiology (*n* = 55; 20.7%), and vascular surgery (*n* = 49; 18.4%). Neurointerventional radiology had the highest mean K_a,r_ (7,799 mGy) followed by neurosurgery (7452 mGy), vascular surgery (6849 mGy), and interventional radiology (6109 mGy). Neurosurgery had the highest mean fluoroscopy time among the medical disciplines (99 min), followed by neurointerventional radiology (90 min), vascular surgery (73 min), and interventional radiology (51 min).

**Table 2:**
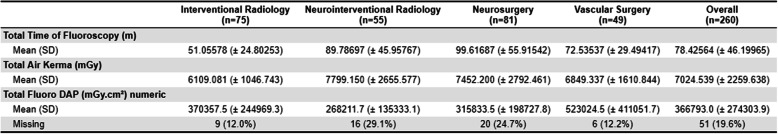
Distribution of high dose procedures by medical discipline and the mean FT, DAP, and K_a,r.._ Statistical significance level is *P* < 0.05 (FT = fluoroscopy time, DAP = dose area product, K_a,r._ = reference point air kerma)

**Fig. 1:**
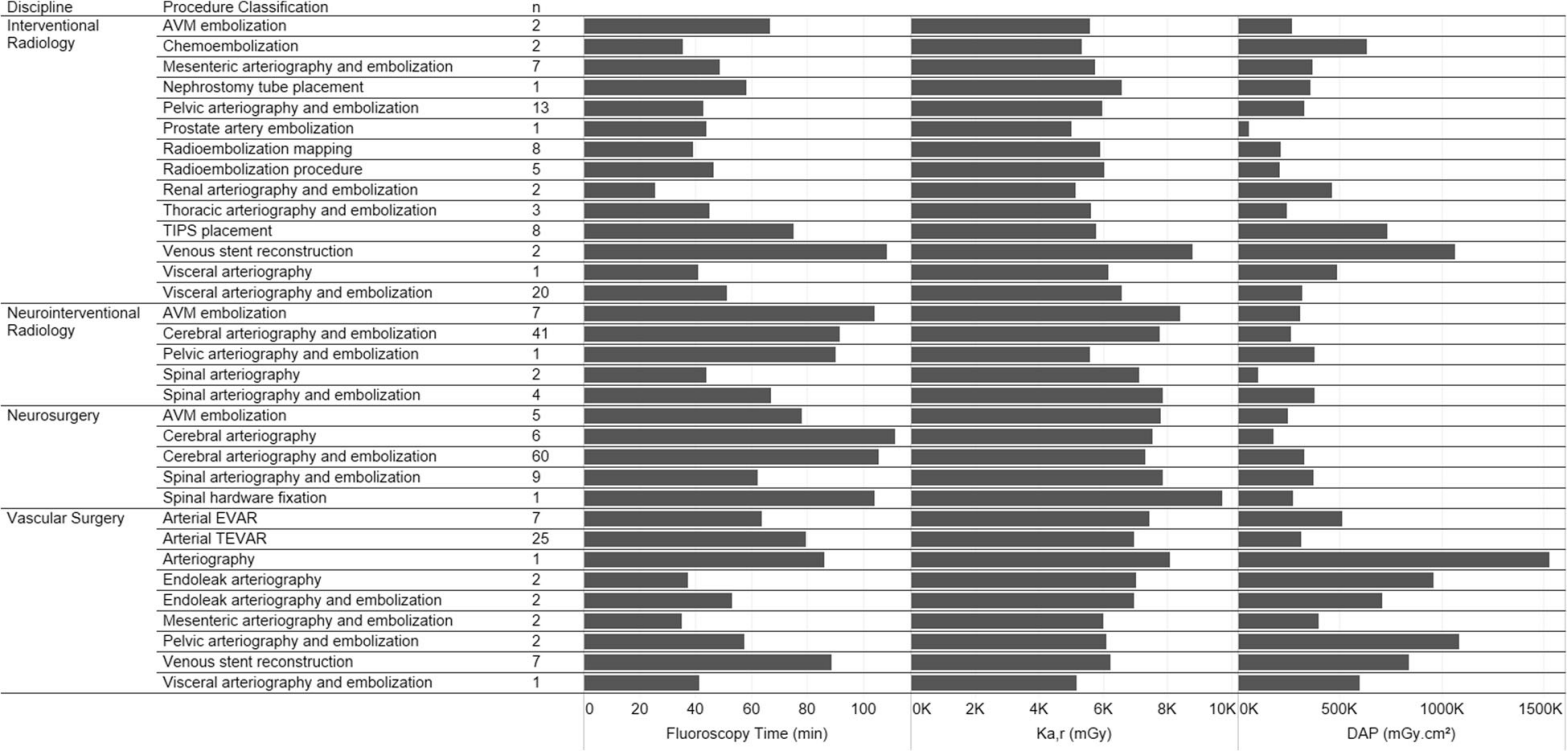
Mean FT, DAP, and K_a,r_ distribution by intervention type and medical discipline (FT = fluoroscopy time, DAP = dose area product, K_a,r._ = reference point air kerma, AVM = arteriovenous malformation, TIPS = transjugular intrahepatic portosystemic shunt, EVAR = endovascular aneurysm repair, TEVAR = thoracic endovascular aortic repair)

Figure 1 displays the radiation dose data distribution by intervention type and medical discipline. The interventions associated with the highest K_a,r_ were venous stent reconstruction performed by interventional radiology, arteriovenous malformation (AVM) embolization performed by neurointerventional radiology, spinal hardware fixation by neurosurgery, and arteriography performed by vascular surgery. The interventions associated with the highest fluoroscopy times were venous stent reconstruction performed by interventional radiology, AVM embolization performed by neurointerventional radiology, cerebral arteriography and embolization performed by neurosurgery, and spinal hardware fixation by neurosurgery. The interventions associated with the highest DAP were venous stent reconstruction performed by interventional radiology, arteriography performed by vascular surgery, and pelvic arteriography with embolization performed by vascular surgery.

**Fig. 2:**
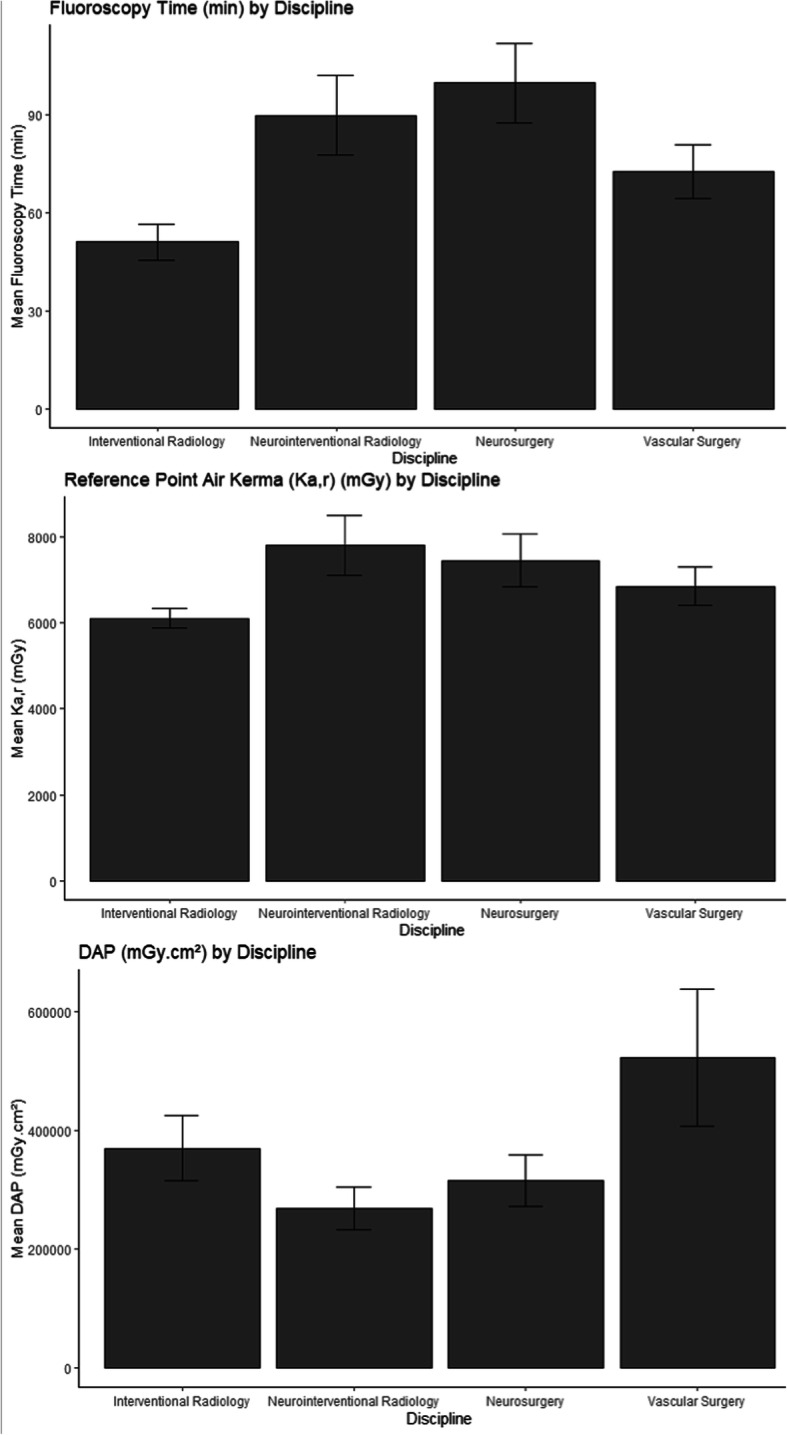
Mean FT, DAP, and K_a,r_, and standard deviation by medical discipline (FT = fluoroscopy time, DAP = dose area product, K_a,r._ = reference point air kerma)

Figure 2 and Table 3 display the mean radiation dose data and standard deviation by medical discipline and the *P* values from radiation dose data comparisons between medical disciplines, respectively. The mean K_a,r_ for interventional radiology performed procedures exceeding 5000 mGy was significantly lower than that for neurointerventional radiology (*p* < 0.001), neurosurgery (*p* < 0.001), or vascular surgery (*p* = 0.02) performed procedures. The mean FT for interventional radiology performed procedures exceeding 5000 mGy was significantly lower than that for neurointerventional radiology (*p* < 0.001), neurosurgery (*p* < 0.001), or vascular surgery (*p* = 0.014) performed procedures. The mean DAPs for neurointerventional radiology and neurosurgery performed procedures exceeding 5000 mGy were both significantly lower than that for vascular surgery performed procedures (*p* = 0.015 and *p* = 0.039, respectively).

**Table 3:**
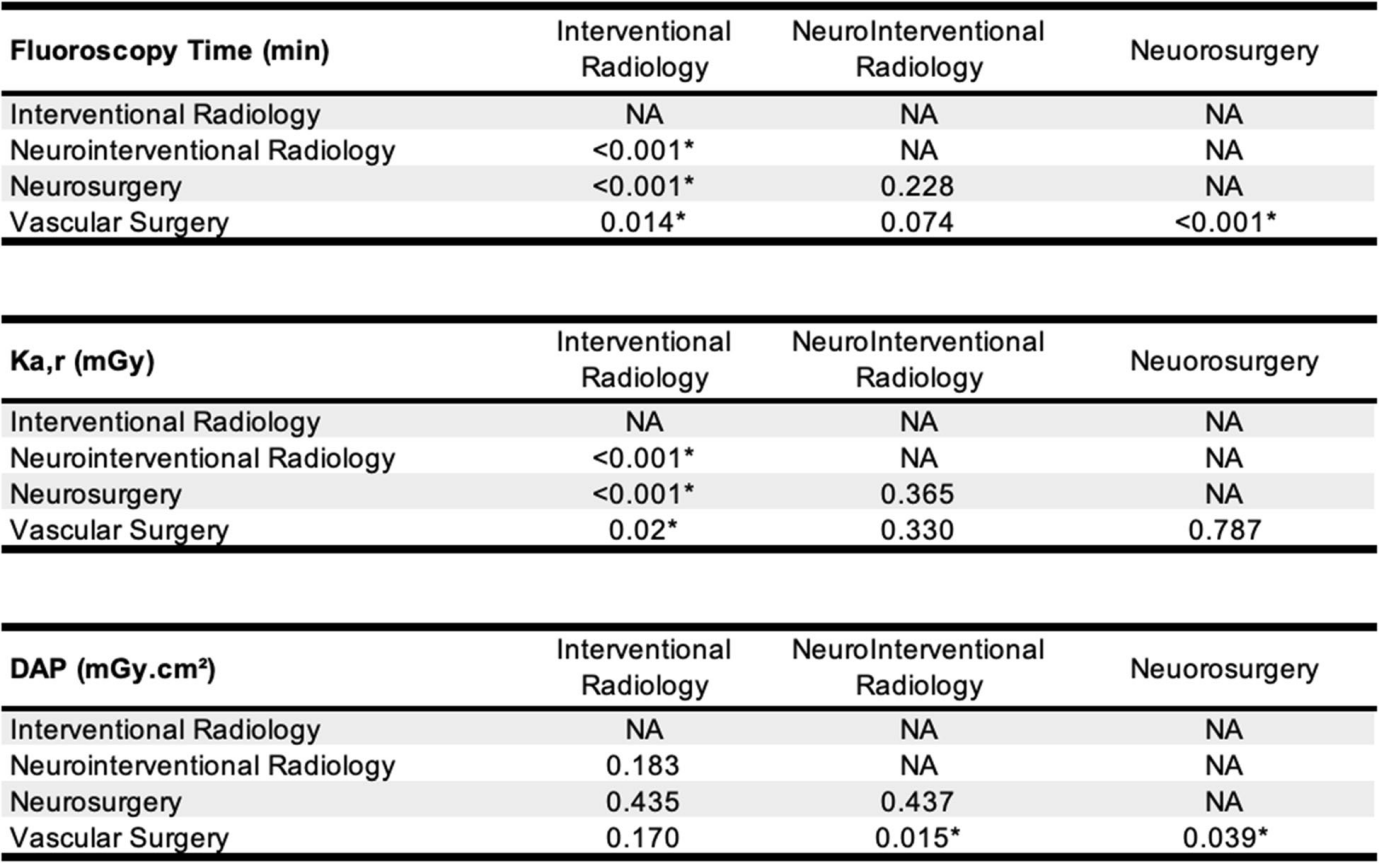
*P* values for comparisons of radiation mean dose data between medical specialties for high dose (> 5000 mGy) procedures. *Statistical significance level is *P* < 0.05 (FT = fluoroscopy time, DAP = dose area product, K_a,r._ = reference point air kerma)

## Discussion

A wide variety of endovascular procedures across many medical disciplines have documented K_a,r_ exceeding 5000 mGy, representing a proportion of procedures which reach a significant radiation dose. These findings comprehensively summarize the specific fluoroscopically-guided procedures which most commonly exceed the threshold radiation dose and are supported by previous studies: Fluoroscopically-guided fenestration, endovascular aneurysm repair, pedicle screw placement, transforaminal lumbar interbody fusion, vertebral augmentation, and AVM embolization have all been previously associated with the highest level of radiation doses (Kirkwood et al. 2015; Srinivasan et al. 2014; Riabroi et al. 2018). Of note, the mean K_a,r_ and FT among all procedures performed by interventional radiology were significantly lower than those performed by neurointerventional radiology, neurosurgery, and vascular surgery. This study demonstrated a significant difference in radiation dose metrics between medical disciplines performing fluoroscopically-guided procedures. While the implications of these findings are not fully elucidated, it may imply that formal standards for radiation dose reduction, such as those put forth by the *American College of Cardiology* and *SIR*, have led to improved radiation safety practices (Stecker et al. 2009; Hirshfeld et al. 2018).

The *RAD-IR* study performed by *Miller* et al analyzed dosimetry data from a variety of interventional radiology and neurointerventional radiology procedures and proposed radiation reference levels for fluoroscopically-guided procedures (Miller et al. 2003; Miller et al. 2009). Their work was aimed at creating mean radiation dose thresholds that when exceeded, could prompt investigation into institution fluoroscopy equipment, procedure protocols, and operator technique to identify areas for improved radiation safety (Miller et al. 2009). Universal radiation dose reference values for all fluoroscopically-guided procedures would provide a means for individual institutions to oversee radiation safety and ensure that interventionalists across medical disciplines are practicing within the expected dose limits for the corresponding procedures.

While medical specialties performing fluoroscopy-guided procedures generally attempt to adhere to the radiation reduction principle of ALARA (as low as reasonably achievable), universal policies regarding patient follow-up when a significant radiation dose is reached are needed to optimize patient care (Hertault et al. 2015; Bartal et al. 2014). The post-procedural recommendations made by *SIR* when a significant radiation dose threshold is exceeded include: documentation in the patient’s medical record, clinical follow-up to assess for deterministic radiation-induced injury, providing written radiation follow-up instructions on the patient’s discharge instruction sheet, and procedural review by a qualified medical physicist (Stecker et al. 2009). The results of this study demonstrate that all physicians performing fluoroscopically-guided procedures may expect to exceed significant radiation dose thresholds occasionally. As such, structured, institution-wide post-procedural policies should be adopted to ensure adequate patient follow-up. *Perry* et al., for example, demonstrated the feasibility of a dose monitoring process utilizing software monitoring and documentation to alert physicians when procedures exceed K_a,r_ of 5000 mGy so clinical follow-up could be arranged to assess for skin injury (Perry et al. 2019). Particularly, all physician groups who perform the procedures that have shown to exceed significant dose thresholds should have an instituted failsafe method for the detection of high doses cases and post-procedural evaluation of the patients with a feedback loop to radiation safety officer/medical physicist after clinical evaluation and patient education.

Similarly, this study provides insight regarding the distribution of specific fluoroscopically-guided procedures which most commonly exceed significant radiation doses. It is critical to consider the adverse health risks associated with occupational radiation exposure and the cumulative impact of small radiation doses obtained during the course of a physician’s career. The cumulative radiation risks include premature cataract formation, early carotid atherosclerosis, and possibly left-sided brain malignancies (Roguin et al. 2013; Ciraj-Bjelac et al. 2010). With this in mind, more aggressive radiation safety practices may be used when performing the procedures listed in Fig. 1 to reduce physician and patient radiation dose. Specific steps which may be taken by interventionalists to reduce patient and operator dose include the use of radiation-absorbing pads, which have been demonstrated to reduce physician radiation dose by approximately 70% during procedures using femoral artery access (Miller et al. 2017; Fetterly et al. 2011). Further, utilization of real-time image noise-reduction technology have demonstrated significant reductions in radiation doses across interventional radiology, cardiology, and neurointerventional radiology-performed interventions (Söderman et al. 2013). Additionally, precisely adjusting collimator boundaries to the region of interest and limiting magnification modes may decrease the contribution of fluoroscopy to the overall radiation dose (NCRP Report 168 | NCRP | Bethesda, MD n.d.). Finally, utilizing the “last image-hold” feature and intermittent, pulsed fluoroscopy on lower frame rates are additional techniques which can minimize both patient and operator radiation doses. One further factor that should be considered regarding increased radiation safety practices is the complexity of the procedure to be performed. While some procedures may exceed significant dose thresholds owing to patient or case specific limitations, other procedures are inherently more complex and will have increased radiation dose exposure regardless of the case specifics (Bundy et al. 2018).

This study has limitations including the single-center, retrospective design of the analysis. Peak skin dose was not directly assessed in this current study; however, *SIR* recommends that K_a,r_ be used as the preferred best clinical approximation of skin dose (Stecker et al. 2009). Image magnification, which affects dose, was not recorded in dose management software and therefore not included in this study. Additionally, the procedures were categorized by MedWatch, the U. S Food and Drug Administration safety reporting program, which does not provide a detailed description of each individual procedure (FDA 2019). Finally, the current study only represents the experiences of physicians from a single-center, therefore potentially limiting the generalizability to other regions.

This study demonstrates that fluoroscopically-guided procedures with high radiation dose exceeding 5000 mGy reference point kerma are uncommon. The majority of cases exceeding 5000 mGy were performed by non-radiologists, who may not receive the same training in radiation physics, radiation biology, and dose reduction techniques as radiologists. This may provide an opportunity for radiology societies to reach out to other medical specialties which perform fluoroscopically-guided procedures to educate and collaborate on radiation safety and establish a multidisciplinary institutional database to ensure consistent follow-up for all high dose cases.

## Conclusions

Fluoroscopically-guided procedures with radiation dose exceeding 5000 mGy reference point air kerma are uncommon. The results of demonstrate that a large proportion of cases exceeding 5000 mGy were performed by non-radiologists, who likely do not receive the same training in radiation physics, radiation biology, and dose reduction techniques as radiologists.

## Data Availability

The datasets generated and/or analysed during the current study are not publicly available, but are available from the corresponding author on reasonable request.

## Abbreviations

FT: Fluoroscopy time
DAP: Dose-area-product
P_KA_: Kerma-area-product
K_a,r_: Reference point air kerma
SIR: Society of interventional radiology
AVM: Arteriovenous malformation
ALARA: As low as reasonably achievable
